# Updates in the Management of Diabetic Macular Edema

**DOI:** 10.1155/2015/794036

**Published:** 2015-04-23

**Authors:** Christopher Mathew, Anastasia Yunirakasiwi, Srinivasan Sanjay

**Affiliations:** ^1^Yong Loo Lin School of Medicine, National University of Singapore, Singapore 117597; ^2^Department of Ophthalmology and Visual Sciences, Khoo Teck Puat Hospital, 90 Yishun Central, Singapore 768828

## Abstract

Diabetes mellitus is a chronic disease which has multiple effects on different end-organs, including the retina. In this paper, we discuss updates on diabetic macular edema (DME) and the management options. The underlying pathology of DME is the leakage of exudates from retinal microaneurysms, which trigger subsequent inflammatory reactions. Both clinical and imaging techniques are useful in diagnosing, classifying, and gauging the severity of DME. We performed a comprehensive literature search using the keywords “diabetes,” “macula edema,” “epidemiology,” “pathogenesis,” “optical coherence tomography,” “intravitreal injections,” “systemic treatment,” “hypertension,” “hyperlipidemia,” “anemia,” and “renal disease” and collated a total of 47 relevant articles published in English language. The main modalities of treatment currently in use comprise laser photocoagulation, intravitreal pharmacological and selected systemic pharmacological options. In addition, we mention some novel therapies that show promise in treating DME. We also review systemic factors associated with exacerbation or improvement in DME.

## 1. Introduction

Diabetes mellitus is a widely prevalent chronic condition that can lead to sight-threatening complications such as diabetic macular edema (DME), proliferative diabetic retinopathy, retinal artery/vein occlusions, and retinal detachment [1]. We would like to discuss the epidemiology, pathogenesis, classification, and risk factors as well as management options for DME.

## 2. Materials and Methods

A comprehensive literature search was conducted on Medline, PubMed^®^, Google  Scholar™, and Cochrane^®^ databases using the keywords “diabetes,” “macula edema,” “epidemiology,” “pathogenesis,” “optical coherence tomography,” “intravitreal injections,” “systemic treatment,” “hypertension,” “hyperlipidemia,” “anemia,” and “renal disease.” Only studies with abstracts and full-texts published in English were included. A hierarchical approach was adopted when selecting articles; relevant articles were initially selected based on their titles and abstracts. The full-texts of these articles were then obtained and reviewed in more detail. 47 studies were eventually collated, comprising 20 clinical trials, 9 review articles, 5 case series/reports, 6 retrospective studies, and 7 prospective studies, published between 1985 and 2014. We chose this time period in order to include seminal papers about the initial gold-standard treatment for DME, laser photocoagulation, especially that of the ETDRS study.

### 2.1. Definition and Classification of Diabetic Macular Edema

Macular edema is defined as retinal thickening or hard exudates at or within 1 disc diameter of the macula centre [2]. Diabetic macular edema is most commonly classified into either being clinically significant or not. Clinically significant macular edema (CSME) is defined as DME meeting at least one of the criteria [2] presented as follows.

*Criteria for Diagnosis of Clinically Significant Macular Edema*

 Thickening of the retina at or within 500 *μ*m of the center of the macula.
 Hard exudates at or within 500 *μ*m of the center of the macula, if associated with thickening of adjacent retina (not counting residual hard exudates remaining after disappearance of retinal thickening).
 Any zone(s) of retinal thickening 1 disc area or larger, any part of which is within 1 disc diameter of the center of the macula.

Diabetic macular edema may also be classified based on optical coherence tomography (OCT) measurements, specifically, thickness of the macula, morphology of the retina, and the presence of macular traction. The latter refers to traction caused either by vitreomacular or epiretinal membranes. Using OCT, DME can be classified into four main types [3] presented as follows.

*OCT Classification of Diabetic Macular Edema*

 
*Type 1*: Early diabetic macular edema.
 
*Type 2*: Simple diabetic macular edema.
 
*Type 3*: Cystoid diabetic macular edema: 3a, mild; 3b, intermediate; 3c, severe.
 
*Type 4*: Serous macular detachment.

Fluorescein angiography (FA) is another modality used to classify DME, into three types [4] presented as follows.

*Fluorescein Angiography Classification of Diabetic Macular Edema*

 
*Focal leakage*: localized areas of leakage from microaneurysms or dilated capillaries.
 
*Diffuse leakage*: diffuse leakage involving the entire circumference of the fovea.
 
*Diffuse cystoid leakage*: mainly diffuse leakage, but accumulation of the dye within the cystic areas of the macula during the late phase of the angiogram.

Fluorescein angiography can also be used to assess the outline characteristics of the foveal avascular zone (FAZ), as reported in the Early Treatment Diabetic Retinopathy Study group report 11 [5]. This is presented in Table 1.

Furthermore, FAZ size has been shown to have no significant correlation with OCT findings such as retinal thickness or volume [6].

### 2.2. Epidemiology of DME

Diabetic retinopathy occurs in 1 out of 3 people with diabetes, with reported rates of DME reaching 7% in this group of patients [7]. In fact, DME is the leading cause of visual loss and legal blindness in people with diabetes [7].

### 2.3. Pathogenesis of DME

Vascular endothelial growth factor (VEGF) is believed to be a key mediator in the pathogenesis of DME. It promotes angiogenesis and causes a breakdown in the BRB by damaging the tight junctions between retinal endothelial cells [8]. These tight junctions are critical to the function and regulation of the BRB [4]. The breakdown of BRB then results in accumulation of plasma proteins such as albumin which exert a high oncotic pressure in the neural interstitium, leading to interstitial edema. Other comorbidities such as chronic hyperglycemia, hypertension, and hyperlipidemia are also implicated in the development of DME [9].

The various treatment modalities for DME can be divided into ocular and systemic forms of therapy.

#### 2.3.1. Ocular Treatments

**Figure 1:**
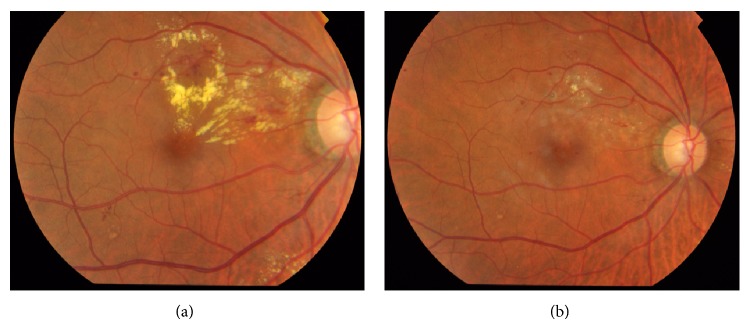
CSME (a) before and (b) after focal laser photocoagulation.

*Laser Therapy*. Focal and/or grid macular laser photocoagulation (MLP) has long been seen as the gold standard for treatment of DME. The grid laser destroys photoreceptors in the retina thereby decreasing the oxygen demand, while the focal laser targets specific leaking microaneurysms responsible for the macular edema. Laser is still the mainstay of treatment for DME [10], although other modalities of treatment are evolving.  Figure 1 shows clinically significant macular edema in the right eye color fundus photograph and after laser photocoagulation.

With increasing research, newer forms of laser are being developed which could minimize the side effects of traditional laser. For instance, subthreshold micropulse diode (SDM) laser may have comparable outcomes, with the added benefit of decreasing the likelihood of scarring [10]. Selective retinal therapy is another selective form of laser that has also shown promise [11].

In recent years, novel treatment modalities that target DME via other mechanisms have been developed. These offer alternative options for treatment of DME refractory to conventional laser therapy. In fact, some have even been used in conjunction with laser, with surprisingly good results. We discuss some of these treatment modalities below.

*Intravitreal Anti-VEGF Therapy*. While the exact pathogenesis of DME has not been completely elucidated, VEGF appears to play a significant role. It is believed to increase pathological angiogenesis as well as the permeability of the BRB, resulting in increased fluid and swelling at the macula. Thus, anti-VEGF therapy could hold a lot of promise in treating DME via inhibition of VEGF. Furthermore, those that specifically inhibit the VEGF-A isoforms might be able to specifically target the pathological angiogenesis occurring in the eye, without affecting physiological angiogenesis throughout the rest of the body [12].

Ranibizumab and bevacizumab are anti-VEGF agents that bind to all VEGF isoforms and fragments, although the latter's use for DME is currently off-label. Aflibercept, the latest newcomer to the market, is a recombinant protein that also binds all VEGF isoforms and fragments. Pegaptanib sodium (Macugen) is an RNA aptamer that selectively binds the VEGF-165 isoform, believed to be the main isomer responsible for DME [13].

**Figure 2:**
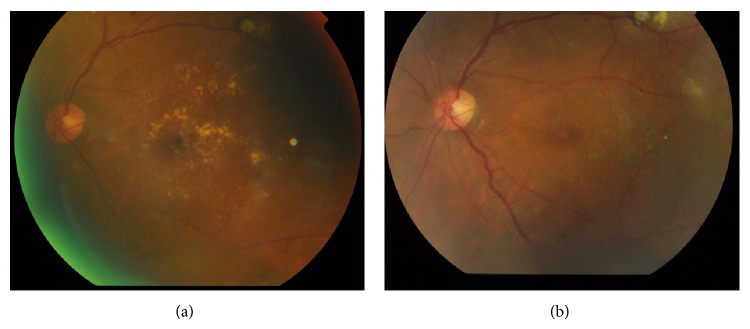
Diabetic macular edema (a) before and (b) after injection of bevacizumab.

A study comparing intravitreal bevacizumab to laser therapy (*BOLT study*) for treating DME showed that, at 2 years, bevacizumab had better outcomes in terms of gain in mean best corrected visual acuity (BCVA) and reduction in central macular thickness (CMT) as compared to laser. In fact, there was a drop in mean BCVA using laser alone [14]. This shows that while laser can effectively reduce anatomical derangements in DME, anti-VEGF agents might have the added benefit of improving functional outcomes (Figure 2).

A double-blind RCT comparing pegaptanib to placebo showed better final BCVA, mean central thickness, and total macular volume in the pegaptanib arm at 36 weeks. The requirement for photocoagulation in eyes treated with pegaptanib was also half that seen with placebo [12]. In terms of functional outcome, pegaptanib also resulted in improved stability during fixation, macular sensitivity, and color discrimination, with the latter two showing positive correlation with the decrease in foveal thickness (FTH) [13]. Again, this shows the benefits of anti-VEGF agents in improving functional outcomes.

The ranibizumab for edema of the macula in diabetes (*READ-2*) randomized, controlled trial (RCT) compared ranibizumab alone, laser alone, and combination therapy for DME and at 6 months after treatment, ranibizumab resulted in a significant improvement in BCVA. Between 2 and 3 years after treatment, there were still significant improvements in mean BCVA and FTH [15]. The effectiveness of ranibizumab might be explained by its ability to strongly suppress aqueous VEGF levels, which has been shown to last for an average of 33.7 days, and even up to 16 months. Furthermore, the extent of suppression does not appear to be affected by baseline VEGF levels [16].

A phase III, multicentre RCT comparing the outcome when anti-VEGF treatment was followed by laser within 10 days or delayed for at least 6 months, showed that the latter protocol resulted in a larger mean increase in BCVA. However, the final central subfield thickness (CST) was comparable between the two groups [17]. This phenomenon could be explained by the potentiation of the laser treatment, after the retina has been sufficiently thinned using anti-VEGF agents. Studies testing the efficacy of laser after anti-VEGF therapy have shown that once the retina has been sufficiently thinned with anti-VEGF drugs, laser photocoagulation does stabilize the retinal thickness and reduce treatment burden. However, reduction in CRT only decreased as long as anti-VEGF injections were being given [18]. Hence, in treating DME, consideration should be paid to deferment of laser therapy after sufficient thinning of the retina with anti-VEGF therapy. While this may not result in further decrease in CRT, it might still improve functional outcomes and reduce the need for further anti-VEGF injections.

However, anti-VEGFs may have their disadvantages, especially in terms of anatomical outcomes. Macular swelling is likely to recur after intravitreal anti-VEGF, requiring repeat retreatments. This is more likely with bevacizumab, given its relatively short intravitreal half-life [19]. A possible alternative could be the use of laser to target leaking microaneurysms after the administration of bevacizumab, which has been shown to result in greater improvements in BCVA and CRT [20], as well as reduce the number of injections needed [18].

Intravitreal bevacizumab has also been reported to cause FAZ enlargement [21] and deterioration of posterior vitreous detachment (PVD) [22], although these appear to be more than offset by the improvement in functional outcomes.

*Intravitreal Steroid Therapy*. Steroid agents are also increasingly being used for the treatment of DME. Triamcinolone, fluocinolone, and dexamethasone are some examples and can come in the form of intravitreal injections or implants.

Studies comparing intravitreal triamcinolone acetonide (IVTA) and MLP show better BCVA and OCT outcomes for IVTA during the first few months [23–25]. However, these findings tend to be reversed by 2 years after initiation of treatment. By 3 years, laser actually results in a greater mean BCVA increase than IVTA. A greater proportion of eyes also show resolution of macular thickening (i.e., CST < 250 *μ*m) with laser than with IVTA [23, 24]. These results suggest that while IVTA may offer significant benefits over laser, these may not last longer than a few months.

However, IVTA may still have a place in treating DME refractory to MLP. Studies evaluating the effect of a single IVTA injection in such cases have resulted in 38% reduction in mean macular thickness at 6 months. Unfortunately, this was also accompanied by significant increases in intraocular pressure (IOP) [26].

Intravitreal steroids can also be administered in the form of implants, which have the advantage of sustained release of the drug. An intravitreal fluocinolone acetonide implant has been shown to result in better outcomes as compared to MLP. At 2 years after implantation, a greater proportion of eyes had ≥15-letter increase in BCVA and significant resolution of macular thickening. However, at 3 years, the results were comparable to those with laser [19]. Once again, it appears that the superiority of intravitreal steroids does not last longer than a few years.

The bevacizumab versus intravitreal dexamethasone for diabetic macular edema (*BEVORDEX*) study compared the efficacy of intravitreal bevacizumab with an intravitreal dexamethasone implant. At 12 months, the two treatments resulted in similar visual acuity outcomes while the key differences were that the intravitreal dexamethasone implant resulted in significantly greater reduction in CMT with the need for far fewer injections [27].

Another clinical trial comparing intravitreal dexamethasone implant in eyes with DME refractory to intravitreal bevacizumab resulted in a significant reduction in CMT and improvement in BCVA up to 3 months after implantation. However, the changes were no longer significant at 4 months [28]. This was reflected in another RCT which showed comparable CMT reduction at 1 year for IVTA with laser and ranibizumab with laser. But once again, there was worsening of macula edema and BCVA in the second year of follow-up for IVTA with laser, unlike the ranibizumab group [29]. These findings indicate that while intravitreal steroid therapy may be at least as good as anti-VEGF therapy, the benefits may not persist in the long term.

If steroid therapy is used, combination with laser may augment the benefits. IVTA with laser has been shown to be comparable to ranibizumab with laser. However, this only held true when limited to pseudophakic eyes, suggesting that this particular combination therapy is best suited to pseudophakic eyes [29].

As mentioned above, anti-VEGF agents may cause worsening of PVD, which is positively correlated with the reduction in CMT. However, this is not seen with IVTA [22]. Hence, steroid therapy may have an advantage over anti-VEGF therapy in cases of DME with preexisting PVD.

Disadvantages of steroids are due to their side effects and occur in a dose-dependent fashion. Studies comparing different doses of IVTA have shown that, with increasing doses, the incidence of IOP elevation increases concomitantly [23]. This increases the need for additional antiglaucoma therapy, which appears to effectively and promptly control the IOP [26].

Another significant effect of steroid therapy is worsening of cataracts. Many studies using IVTA or intravitreal FA have reported the eventual need for cataract extraction in up to 100% of eyes undergoing steroid therapy [28, 30].

*Intravitreal NSAID Therapy*. Intravitreal nonsteroidal anti-inflammatory drugs (NSAIDs) have also shown good results in treating DME. NSAIDs block prostaglandin synthesis and reduce inflammation, which may have a role in macular edema. Certain nonselective NSAIDs such as diclofenac also inhibit lipooxygenase synthesis mimicking the method of action of steroids, which may explain their similar efficacies. In fact, intravitreal diclofenac has showed comparable reductions in CMT and BCVA improvement as IVTA. In addition, the diclofenac had the added benefit of reduced IOP, as opposed to increased IOP with the triamcinolone. Hence, it appears that intravitreal diclofenac could possibly be as effective as steroid therapy, while avoiding the related adverse effects such as IOP elevation [29].

*Vitrectomy*. Another surgical option used as an adjunct in the treatment for DME is vitrectomy. The removal of the vitreous is believed to reduce vascular permeability and relieve traction on the retina. Indeed, vitrectomy combined with IVTA and MLP for eyes with DME refractory to prior anti-VEGF therapy has resulted in significant improvements in BCVA and CST [31]. DME refractory to previous IVTA therapy has also shown good response once vitrectomy was performed before IVTA was repeated, with significant improvements in BCVA and the rate of DME resolution reaching 77.5% [31].

However, for DME refractory to previous MLP, vitrectomy used as an adjunct therapy has had equivocal results at best [31–35]. Hence, this suggests that vitrectomy for refractory DME should mainly be considered for DME unresponsive to prior anti-VEGF or steroid therapy, but not to prior MLP for which there has been no proven added benefit.

*Other Novel Therapies*. Even more innovative treatments are being developed for DME, comprising both pharmacological and nonpharmacological therapies.

Recently published results of a clinical trial using intravitreal injections of varying doses of a new drug to treat refractory DME have shown promising results. The drug MP0112 is a designated ankyrin repeat protein that selectively binds VEGF-A isoforms. Significant reductions in FTH and improvements in BCVA were seen, with the effects showing dose-dependency. Furthermore, aqueous levels of MP0112 remained detectable after 12 weeks, suggesting a relatively long half-life of the drug [33]. Randomized, controlled trials for this drug are definitely needed to evaluate the possibility of its use to treat DME.

Another recently developed drug is PF-04523655 (PF), an siRNA that binds to and inhibits the RTP801 gene. This gene is responsible for the production of hypoxia-inducible factor which, in turn, regulates VEGF production. The dose-ranging evaluation of intravitreal siRNA PF-04523655 for diabetic macular edema (*DEGAS*) RCT comparing different doses of PF with laser showed that PF resulted in greater BCVA improvements, although CST reduction was only half as good as that seen with laser. The change in BCVA, but not that in CST, showed a positive dose-dependency correlation. Furthermore, there was no apparent increase in toxicity with higher doses of PF [34]. Again, this calls for RCTs to test the efficacy of PF.

A nonpharmacological treatment that has recently been reported is photobiomodulation (PBM). A case series testing the efficacy of daily PBM therapy for 2 months showed a mean decrease in macular thickening of 20%. However, this study only enrolled patients with non-centre-involving DME (NCDME) and, thus, it still remains to be seen if PBM will have similar effects in CDME. In any case, it is thought that there is a significant risk of progression of NCDME to centre-involving DME (CDME) and, hence, there may be a place for PBM therapy yet [35].

Another newly developed local treatment for DME is subtenon injections of interferon-*α* (IFN*α*). This drug acts as an inhibitor of VEGF and other cytokines and also enhances the BRB. So far, it has only been tested in a single case report [36], with good results, and more studies should definitely be done to evaluate the effectiveness of this treatment.

Other than the local treatment methods mentioned above, other systemic therapies have shown positive results with regard to DME.

#### 2.3.2. Systemic Treatments

*Fenofibric Acid Therapy*. Considering that poor control of total cholesterol and LDL cholesterol may worsen DME [37], it stands to reason that pharmacotherapy for dyslipidaemia is likely to have beneficial effects on DME. Indeed, one review article reported a 31% reduction in the need to start laser treatment for both DME and PDR with systemic fenofibrate. Also, the addition of fenofibrate to simvastatin decreased the risk of progression of retinopathy by 40% [30].

However, an RCT (*MacuFen study*) comparing 135 mg daily fenofibric acid with placebo did not find any significant changes in BCVA or total macular volume (TMV) at 12 months [38]. A possible explanation might be that since there was no significant change in total cholesterol levels in this study, the dosage of the fenofibric acid might not have been sufficient to improve the DME. Yet another reason, as suggested by the authors themselves, might be the fact that the study was underpowered to detect any significant beneficial effect of fenofibric acid. Hence, it appears that further adequately powered studies are needed to determine whether fenofibric acid could improve DME, and if so, at what dose.

*Systemic Erythropoietin Therapy*. Patients with end-stage renal failure (ESRF) tend to have anaemia due to the impaired production of erythropoietin (EPO) by the kidneys. Surprisingly, there have been reports of improvement in DME in patients with ESRF treated with subcutaneous EPO injections. This was supported by the findings of a case series in which 3 diabetic patients with existing diabetic retinopathy (DR) were treated with subcutaneous EPO injections. After 6–11 months of treatment, not only had their haematocrit levels increased, but they also had improvements in BCVA and the severity of DME [39].

These results are not surprising as studies seem to suggest that EPO may have a protective role in eyes affected by DME. The current literature posits that EPO might enhance the function of the BRB and protect against the damaging effects of VEGF. Comparing macular edema secondary to central/branch vein occlusion and diabetes, vitreous EPO levels in the latter group have been shown to be significantly higher than in the former group. This suggests that instead of being responsible for causing macular edema, EPO may in fact be produced as a result of DME due to its neuroprotective effects [40].

*ACE Inhibitor Therapy*. Given that poor control of blood pressure is another major risk factor for the development and progression of DR, it comes as no surprise that ACE inhibitor therapy does appear to have beneficial effects on DME. However, what is interesting is that these effects are still seen despite the fact that there is no significant change in blood pressure, suggesting that these medications may improve DME via a mechanism other than via lowering of blood pressure [30]. Another study comparing the effect of daily oral captopril with placebo revealed that a significantly higher proportion of patients on captopril had an improvement in BCVA of ≥2 lines, a significant decrease in FTH, and a delay in DR progression. Again, this study reported no significant change in the HbA1c levels throughout the follow-up, removing better control of blood glucose as a confounding factor [41].

It is important to note that there are also treatments that may have negative effects on DME. These range from specific drugs to systemic factors, some of which are explored further below.

*Glitazone Therapy*. Recently, the use of glitazones (in the class of thiazolidinediones) for treating diabetes has been called into question. Despite their effect on blood glucose levels, they have been reported to worsen DME [37]. Some theories as to how these drugs do so include causing fluid overload and increasing plasma VEGF levels. A retrospective study of patients with DME on glitazones showed a reduction in macular edema in 73% of patients over 1 to 2 years after cessation of the glitazones. However, the improvement in visual acuity was less convincing, being reported in only 27% of patients [42]. Nevertheless, it may be worthwhile stopping glitazone therapy if it is indeed found to worsen DME.

*Insulin Therapy*. Another mainstay of diabetes treatment, insulin, has also been shown to possibly cause worsening of DME in the short time period just after it is started, that is, “early worsening” of DME [38]. In fact, a retrospective case series of diabetic patients previously treated with anti-VEGF or MLP for DME, and taking insulin, still had significant improvements in BCVA and CST that were comparable to those taking oral diabetic agents instead [43]. By 4 years after initiation of insulin treatment, insulin also does not seem to cause any significant increase in the risk of progression of DR [30]. Hence, it appears that any adverse effect of insulin on DME may be short-lived and outweighed by the benefits from the tighter blood glucose control with insulin.

*Blood Glucose Levels*. Considering that DME develops as a sequela of diabetes, we would expect an association between poor control of blood glucose levels and deterioration in DME [37].

*Anaemia*. Anaemia is another factor that is thought to worsen DME [37, 44]. In fact, studies have shown that Hb levels <12 g/dL result in doubling of the risk of DR [45]. This might explain why systemic EPO therapy appears to improve DME [37], likely via an increase in haemoglobin levels. This results in increased oxygenation of the retina and, ultimately, less ischemia-induced VEGF production. Another possible mechanism is via the neuroprotective role of EPO on the retina, as mentioned previously.

*Hypertension*. Poor control of hypertension has also been shown to increase the risk of development and progression of DR, as compared to diabetic patients without hypertension [45]. Hence, control of hypertension plays a significant role in the management of DME [37].

*Dyslipidaemia*. Dyslipidaemia has also been associated with worsening of DME. More specifically, better control of LDL-cholesterol levels appears to result in improvement of DME [37]. However, HDL-C levels do not seem to be predictive of diabetic retinal lesions, and the association between total cholesterol levels and DR is equivocal [37, 46].

*Kidney Disease*. Kidney disease has also been shown to cause deterioration of DME. Studies have shown an association between microalbuminuria/nephropathy and DR [44, 45], as well as worsening of DME [37]. This might be explained by the fluid retention secondary to the hypoalbuminaemia caused by kidney disease. It is of note that haemodialysis treatment for ESRF does not seem to have any effect on DME though [47].

*Pregnancy*. An array of physiological changes occur during pregnancy, some of which unfortunately do seem to cause a rapid progression of DR. However, this is more often than not a transient worsening and does not increase the risk of eventual progression of DR in the long term [45].

## 3. Conclusion

DME is a common ocular manifestation of diabetes and has the potential to cause significant visual loss. However, many modalities of treatment have been developed to treat the condition, each with their own benefits and drawbacks. In addition, even more novel therapies have been developed in recent years and show very promising results. Despite the choice of therapy adopted, control of other systemic comorbidities is also important in improving outcomes of treatment. As more research is done on this condition, we hope that even better therapeutic modalities can be uncovered so as to effectively treat DME and ultimately reduce its associated complications.

## Figures and Tables

**Table 1:** Grading of foveal avascular zone using fluorescein angiography. ^*^As defined in the ETDRS group report 11.

Grade	Outline of FAZ	Size of FAZ	Capillary loss
Grade 0	Outline of FAZ normal	Size of FAZ less than the area of the 300 *µ*m radius circle^*^	Capillary loss absent

Grade 1	Outline of FAZ not smoothly round or oval-shaped. Appreciable irregularities seen but not necessarily abnormal	Size of FAZ equal to area of the 300 *µ*m radius circle^*^	Capillary loss questionable

Grade 2	Outline of FAZ obviously damaged but limited to less than half of the circumference	Size of FAZ greater than the 300 *µ*m radius circle^*^ but less than the 500 *µ*m radius circle^*^	Capillary loss definitely present

Grade 3	Outline of FAZ destroyed for at least half the circumference, with some remnants still present	Size of FAZ greater than or equal to the 500 *µ*m radius circle^*^	Moderate capillary loss

Grade 4	Capillary outline totally destroyed		Severe capillary loss

Grade 8	Cannot be graded	Cannot be graded	
